# Basketball Shot Types and Shot Success in Different Levels of Competitive Basketball

**DOI:** 10.1371/journal.pone.0128885

**Published:** 2015-06-03

**Authors:** Frane Erčulj, Erik Štrumbelj

**Affiliations:** 1 Faculty of sport, University of Ljubljana, Gortanova 22, 1000 Ljubljana, Slovenia; 2 Faculty of computer and information science, University of Ljubljana, Večna pot 113, 1000, Ljubljana, Slovenia; National Scientific and Technical Research Council (CONICET)., ARGENTINA

## Abstract

The purpose of our research was to investigate the relative frequencies of different types of basketball shots (above head, hook shot, layup, dunk, tip-in), some details about their technical execution (one-legged, two-legged, drive, cut, …), and shot success in different levels of basketball competitions. We analysed video footage and categorized 5024 basketball shots from 40 basketball games and 5 different levels of competitive basketball (National Basketball Association (NBA), Euroleague, Slovenian 1^st^ Division, and two Youth basketball competitions). Statistical analysis with hierarchical multinomial logistic regression models reveals that there are substantial differences between competitions. However, most differences decrease or disappear entirely after we adjust for differences in situations that arise in different competitions (shot location, player type, and attacks in transition). Differences after adjustment are mostly between the Senior and Youth competitions: more shots executed jumping or standing on one leg, more uncategorised shot types, and more dribbling or cutting to the basket in the Youth competitions, which can all be attributed to lesser technical and physical ability of developing basketball players. The two discernible differences within the Senior competitions are that, in the NBA, dunks are more frequent and hook shots are less frequent compared to European basketball, which can be attributed to better athleticism of NBA players. The effect situational variables have on shot types and shot success are found to be very similar for all competitions.

## Introduction

The non-free-throw basketball shot (or field goal) is the primary way of scoring and one of the most frequent and important technical elements in competitive basketball [1]. Players shoot using different techniques, the choice of which is influenced by several factors, such as distance, angle, player type, etc … In order to be an effective basketball shooter, a player must be trained in choosing the appropriate technique and executing it. And, because practice time is limited, the techniques that have to be utilized more frequently in competition should be practised more frequently as well.

Therefore, as a first step towards improving the quality of the basketball training process, we require a better understanding of which basketball shot techniques are executed more frequently in competition and in which situations. Furthermore, we want to understand how large the differences between age groups and levels of competition are, in particular, between Youth and Senior level or between European basketball and the National Basketball Association (NBA). In such cases we would expect that major differences in athleticism (especially between youth and senior basketball) and overall technical ability, would also have an effect on shot type selection and technique.

While there has been some work on the technical elements of dribbling and passing [2, 3], related work on understanding the basketball shot is based only on officially recorded statistics (shot success and, sometimes, shot location) [2, 4–12]. Game statistics provide us with only limited insight and no information about the technical aspects of the shots. The few properties with respect to the basketball shot that have been explicitly analysed and supported by empirical research are that more successful teams, on average, have fewer three-point attempts and a higher shooting percentage [2, 5, 9] and that guards attempt more shots from long range than centres, especially three-point shots [10, 11]. A very often researched related topic is the hot hand phenomenon (see [13] and references therein).

The lack of directly related work is understandable, because data on the technical execution of basketball shots are not readily available. In recent years, there have been substantial advancements in automated player and ball tracking. Technology implemented in the NBA, is capable of (semi-)automated recognition of such technical elements as shot type and defensive spacing, and these data are already being used in research [13, 14]. For other technical aspects and basketball competitions other than the NBA, researchers currently have no choice but to manually collect the data by visually inspecting games.

In order to gain more insight into basketball shooting in competition, we visually inspected all non-free-throw shots in 40 competitive basketball games from 5 different levels of competition for a total of 5024 basketball shots. For each shot, we recorded several technical features, the situation in which the shot was executed, and whether or not the shot was successful.

Our primary goal was to estimate the relative frequencies of different technical features of the basketball shot and how these frequencies compare across different levels of competition. Furthermore, we examined whether substantial differences in frequency between competitions are due to differences in shot selection or because some situations arise more frequently in some competitions.

## Methods

### Target variables of interest

We focused on the following technical features of a basketball shot:

- **Shot type** We divided shots into the following 5 basic shot-type categories:
  - *above head*: Shooting the ball above the head, looking from under the ball towards the rim. This shot type is the most often used shot-type when shooting from distance, but can also be utilized when near the basket. The most typical example of an above head shot is the jump shot. S1 Video
  - *hook shot*: Shooting the ball turned approximately perpendicular to the basket using the arm facing away from the basket in a sweeping motion, extending the shoulder movement and bending the wrist. Note that half-hook shots (approximately facing the basket) were also categorized as hook shots. S2 Video
  - *layup*: A one-handed shot made by holding the ball from below and releasing it after an upwards motion of the arm. Typically executed near the basket and jumping from one leg. Sometimes executed by bouncing the ball off the backboard. S3 Video
  - *tip-in*: Shooting the ball by tipping a rebound into the basket. This type of shot is entirely executed mid-air. S4 Video
  - *dunk*: Shooting the ball down through the basket with the hands above the rim. This technique is limited to players of sufficient height and/or vertical jump. S5 Video
  Shots that did not fit into these 5 types were left uncategorised and labelled as *other* shot types.
- **Leg position** Shots can be executed standing on (or jumping from) a single leg (*one-legged*) or both legs (*two-legged*). S6 and S7 Videos
- **Movement** Most shots are executed from a stationary position (*no* movement), some are executed after moving towards the basket, which we split into two subcategories: (a) direct unopposed drive (penetration) straight to the basket (*drive*) S3 and S6 Videos and (b) dribbling towards the basket, beating the defender, or being passed the ball while running towards the basket (*dribble or cut*). S8 and S9 Videos

**Fig 1:**
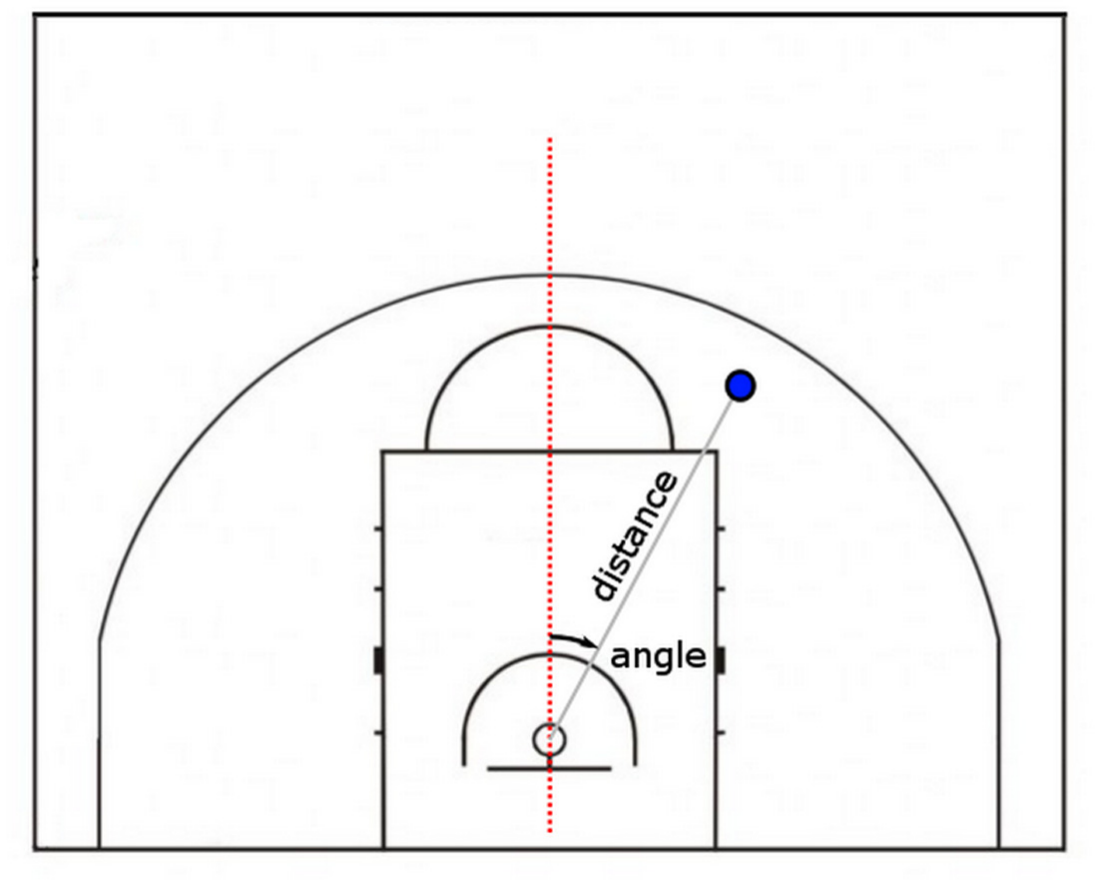
A diagram of how we decomposed shot location into distance and angle. The blue dot represents the location of the basketball shot.

The following situational variables that might influence shot type selection in a given situation were also recorded:

- **Player type** We categorized players using the three major player-types: guard (*G*), forward (*F*), and center (*C*). These player-types have different roles in competitive basketball [15] and they have different morphological (anthropometric) dimensions [11] and motor skills/potential [15, 16]. These differences lead to differences in basketball shooting. One of the main differences is that guards play farther from the basket and more often shoot from distance [10, 17]. Centres on the other hand, play closer to the basket and, due to this and their morphological characteristics, more often execute the dunk or tip-in.
- **Location** The location of the shot on the basketball court with respect to the basket substantially influences shot type selection. We decomposed shot locations into *distance* (in meters) and absolute *angle* (in degrees) components (see Fig 1). That is, we do not distinguish between the left- and right-hand side.
- **Transition** This feature indicates that the shot was made during a transitional attack (fast breaks, secondary breaks, …). That is, while the defending team was still in transition from attacking to defending and not all 5 of their players were in their appropriate defensive positions.

We were also interested in **Shot success**: *made* or *missed*.

### Data acquisition

We analysed games from 5 different levels of competition (3 Senior and 2 Youth competitions):

- **NBA** The National Basketball Association (NBA) is the strongest men’s Senior club basketball competition. It features the best and most paid basketball players from all around the World.
- **EURO** The Euroleague is the top-tier of inter-European men’s Senior club basketball. It features the top clubs from the best European national leagues and therefore the some of the best senior men’s basketball players in Europe and the World.
- **SLO1** The top-tier Slovenian national league ranks in terms of team and player quality in the mid-range of European national competitions. It features Slovenian and foreign Senior players, the majority of whom are professional basketball players, but are not good enough to compete at the international level
- **U16 and U14** The Under-16 and Under-14 Slovenian boys Youth competitions feature the best young Slovenian basketball players. These players are still in the process developing both physically and in terms of basketball knowledge and expertise.

We analysed one full round of the NBA Playoffs and one full round of the Euroleague regular season. The choice of NBA Playoffs instead of the Regular season was deliberate. Due to a very large number of regular-season games in the NBA and a very packed schedule, not all games of the regular season are fully competitive. This is especially true towards the end of the season, when some teams have no motivation to win or even motivation to lose in order to finish lower and earn a better position in the next-year’s player draft. For SLO1, two full rounds of games were analysed. A broader sample was not possible for U16 and U14, because youth basketball games are typically not videotaped. We focused on the final tournament, where the top 4 teams in that season play. For a detailed list of games for each competition see S1 Text.

Every game was analysed by an expert (all experts had playing and coaching experience and were instructed beforehand on how to categorize shots to ensure consistency) who recorded all features of interest for every non-free-throw basketball shot in that game. Every game was analysed by a single expert and every expert analysed several games. For each expert, the aggregate of all games analysed by that expert was re-checked for consistency by another expert and no inconsistencies were found.

For NBA, EURO, and SLO1 games, television broadcast footage was used. For U14 and U16 games, tournament organizers official game footage was used. The data are summarized in Table 1. The complete data set is available for download S1 Dataset.

**Table 1:** Summary of acquired data.

	EURO	NBA	SLO1	U14	U16
games	12	8	10	6	4
teams	24	16	10	4	4
centers	53	27	16	9	11
forwards	98	61	39	24	15
guards	105	70	55	14	14
shots	1371	1228	1182	738	505
shots/game	114.3	^a^153.5	118.2	123.0	126.25
shot success [%]	52.0	53.2	51.4	52.2	55.0
avg. distance [m]	4.12	^b^4.16	4.10	3.05	3.33
avg. angle	50.78	40.08	42.96	45.57	48.80

^(a)^ An NBA game is 48 minutes long, European basketball games are 40 minutes long. NBA game length adjusted shots/game value is 127.9.

^(b)^ The NBA 3-point line is at approximately 7.25m, while the European 3-point line is at 6.75m.

### Statistical analysis

The main objective is to estimating the relative frequencies of outcomes for the following variables: shot type, leg position, movement, and shot success. In addition, we want to compare these estimates across different competitions and to examine if anything changes when relative frequencies are adjusted with situation-based covariates. The features of interest are all categorical variables. We model each of them separately using a Bayesian hierarchical multinomial logistic regression model.

The multinomial model is a natural choice given the categorical variables. We opt for a hierarchical model to facilitate partial pooling. That is, we want to allow the base rates and situational-covariate coefficients to vary across competitions, but we expect them to be similar. Partial pooling also alleviates the problem of comparisons [18].

Let our target variable have *r* different categories *y*_1_, …, *y*_*r*_ and let *Y*_*i*,*j*_ be its value for the *i*–th shot of the *j*–th competition. We model the probabilities as

$$\begin{array}{c} Pr\left(Y_{i,j}=y_{l}\right)=\frac{\exp(\beta_{l,j}X_{i,j})}{\sum_{k=1}^{r-1}\exp\left(\beta_{k,j}X_{i,j}\right)},\;\text{for}\;l=1..\left(r-1\right) \end{array}$$

and for the reference category

$$\begin{array}{c} Pr\left(Y_{i,j}=y_{r}\right)=1-\sum_{k=1}^{r-1}Pr\left(Y_{i,j}=y_{k}\right). \end{array}$$

The model ***β***_*l*,*j*_ ***X***_*i*,*j*_ in the exponent is composed of *m* independent variables and the constant term. We put hierarchical priors on every coefficient:

$$\begin{array}{c} \beta_{i,l,j}∼N\left(\mu_{i,l},\sigma_{i,l}^{2}\right),\;\text{for}\;i=0..m,l=1..\left(r-1\right),j=1..n, \end{array}$$

where *n* is the number of different competitions. We give the hyper-parameters *μ* and *σ* ^2^, weakly-informative priors N(0,1000) and InverseGamma(10^−4^,10^−4^), respectively.

When modelling shot type, leg position, and movement variables, we used angle, location, player type, and transition as independent variables. For shot success, we used all of these seven variables. Some of the independent variables are nominal and we use dummy coding to enter each of them into the regression as *q*−1 binary independent variables, where *q* is the number of categories of that nominal variable. Reference categories for nominal variables are (when dummy-coded or when they are the target variable): above head shot type, one-legged, no drive, no transition, missed, and forward player type.

We’ll refer to the above model as **Model 3**. It is the most general model used in this research, but in most cases, we will use two simplifications:

- **Model 2** is obtained from Model 3 by assuming that the effect of the covariates are the same across all competitions. That is, all competitions have a common set of independent variable coefficients *β*. Each competition still has its own base rates *β*_0_.
- **Model 1** is a further simplification, where we assume that situational-covariates have no effect on the outcome. That is, we only use *β*_0_ coefficients, so Model 1 is equivalent to estimating the relative frequencies for each competition separately.

#### Model fitting, evaluation, and reporting

All models were coded and estimated using the Stan software for Bayesian inference [19]. For each model, we ran a 5000 adaptation iterations and 5000 sampling iterations. Trace plots and convergence diagnostics values did not show any indication of non-convergence. Assuming convergence, Monte Carlo standard errors of all reported values were less than 0.001. The out-of-sample predictive performance of each model for each outcome was estimated using the Widely Applicable Information Criterion (WAIC) [20]. All reported posterior point estimates are means and all confidence intervals cover 95% and are based on the 0.025—0.975 quantile interval.

## Results

The results are split into two parts. In the first part we report the estimates of the relative frequencies of the variables of interest and compare them across competitions. We report two sets of estimates:

1. Estimates using Model 1. These estimates can be used to compare the raw relative frequencies across competitions. They could be used to compare the underlying shot type selection process only if we could assume that all competitions have the same distribution of situations that arise (this is clearly not true; for example, different distance distributions; see Table 1) or that the situation in which the shot was made does not influence the choice of technique or shot success (again, most likely not true).
2. Estimates adjusted for situational variables. These were obtained by fitting Model 2, computing the posterior predictive distributions, and estimating the mean relative frequencies. The Euroleague was used as the common denominator. That is, for each competition, we predicted the relative frequencies for the case where the distribution of situations was as is in the Euroleague.

In the second part, we investigate loosening the assumption that the effect of situational variables is the same for all competitions and allowing for competition-specific but related coefficients (Model 3). In particular, how this improves predictive accuracy.

### Relative frequency estimates

#### Shot type

**Fig 2:**
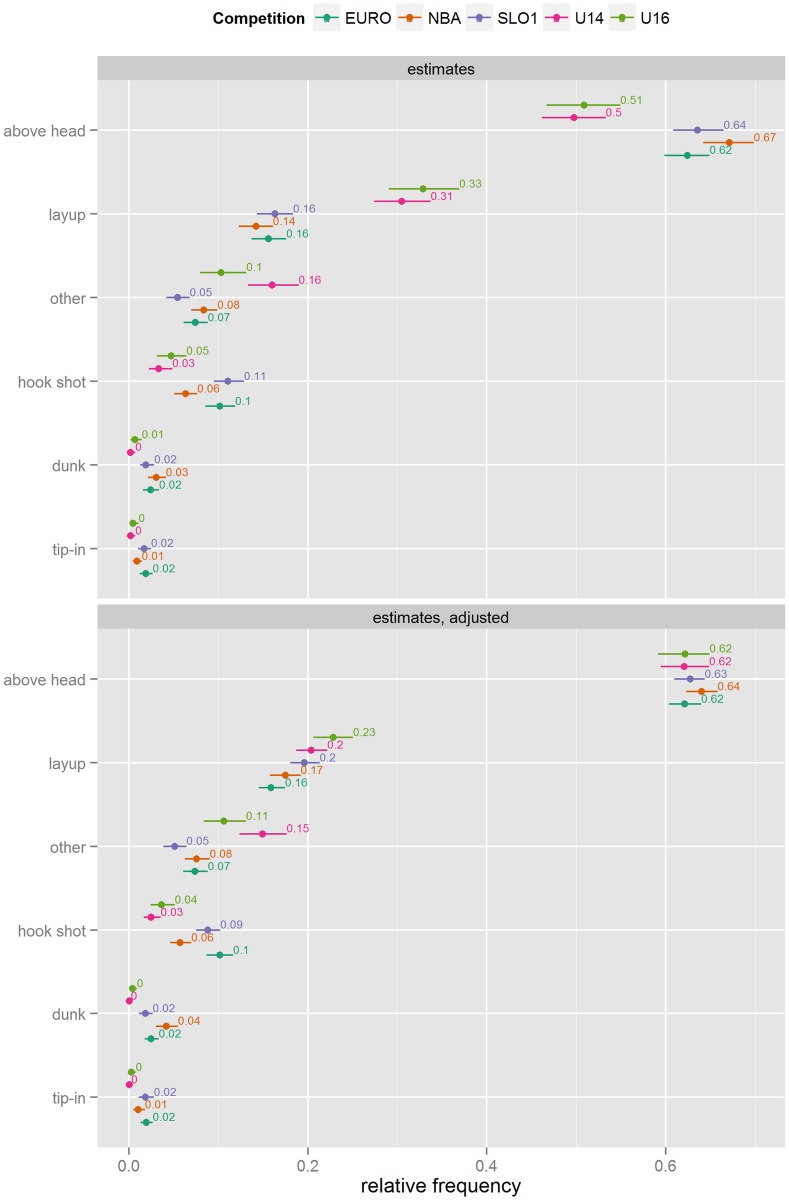
Shot type frequency estimates (unadjusted and adjusted for differences in situational variables).

Overall, the most frequently observed shot type is the above head shot, followed by the layup (see Fig 2). The layup and uncategorised (other) shots are more frequent in Youth basketball competitions, while above head, dunks, tip-ins, and hook shots are less frequent. The only discernible difference between the three Senior competitions is that the hook shot is less common in the NBA, compared to the two European basketball competitions. Adjusting for differences in situations that arise increases the difference in relative frequency of dunks between NBA and other senior competitions and removes the large differences in relative frequencies of above head shots and layups between the Youth and Senior competitions.

#### Leg position

**Fig 3:**
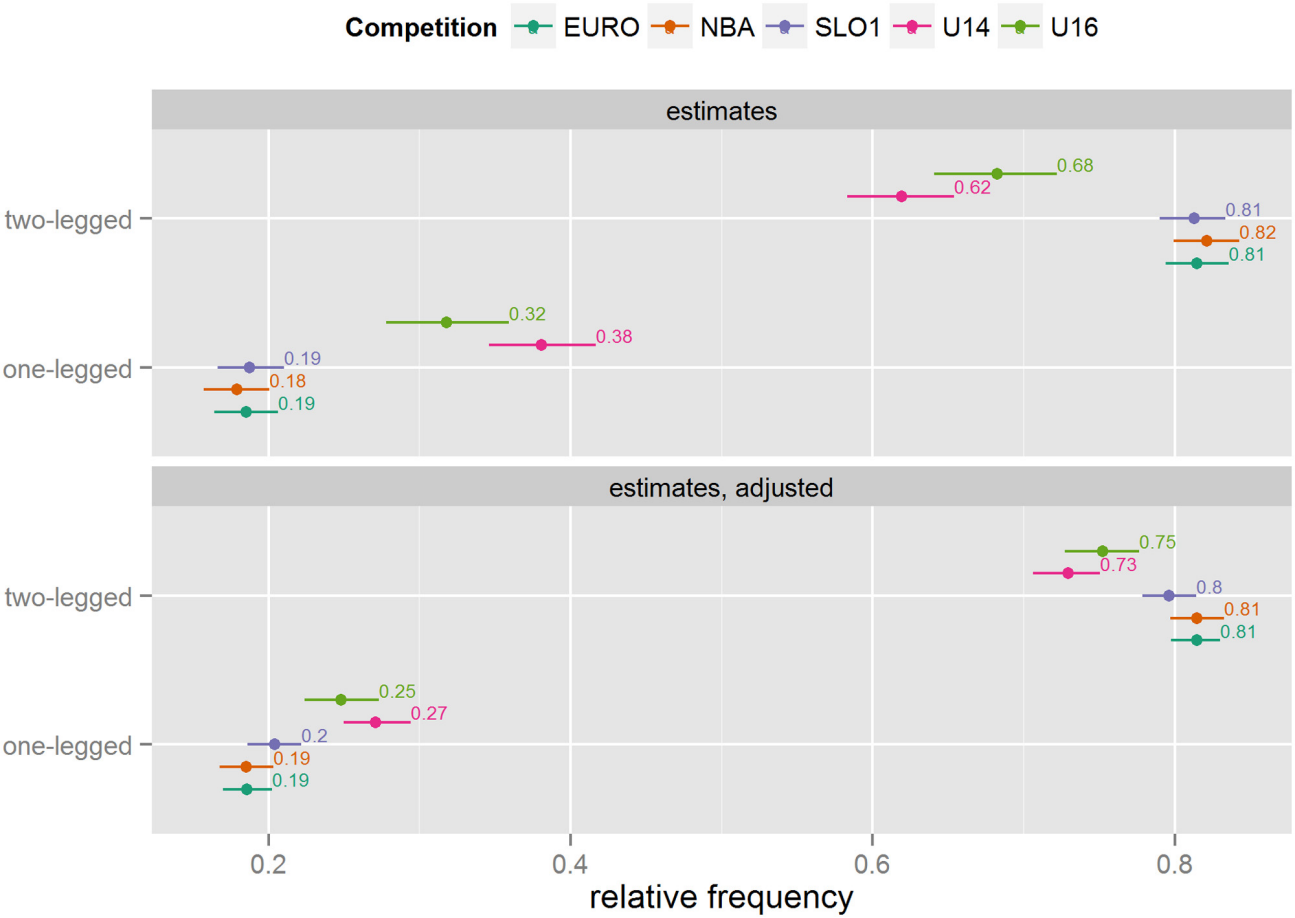
Leg position frequency estimates (unadjusted and adjusted for differences in situational variables).

In senior competitions, approximately four fifths of all shots are attempted standing on or jumping with both legs (see Fig 3). In Youth competitions, this number is about 10% lower, which could be attributed to Youth basketball players using the layup more. However, the differences, although smaller, persist even after adjustment. There are no discernible differences within the Youth or Senior competitions.

#### Movement

**Fig 4:**
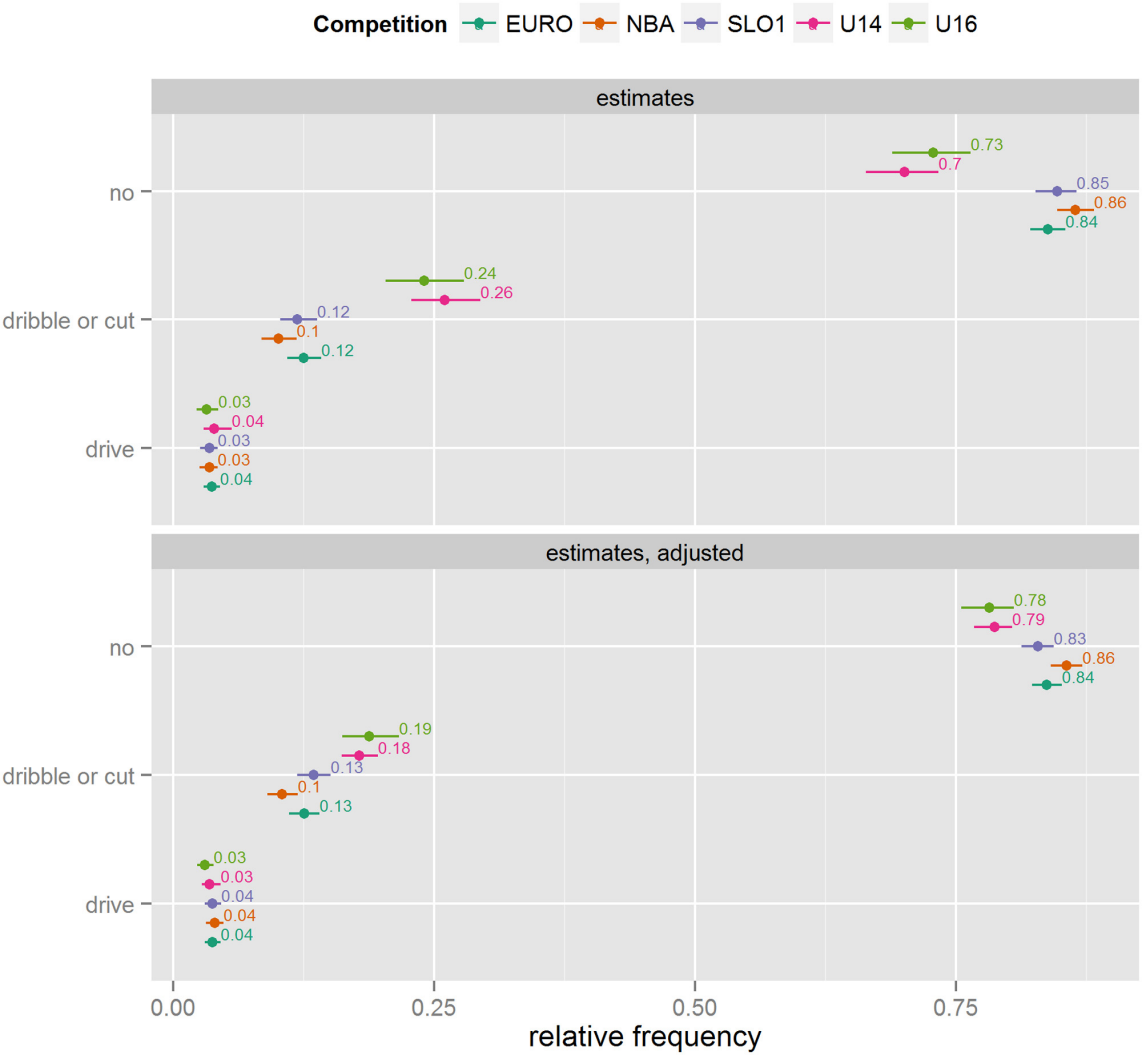
Movement frequency estimates (unadjusted and adjusted for differences in situational variables).

Most shots are attempted from stationary situations, approximately one quarter after the player dribbles or cuts through the defence, and only a small fraction of situations arise where the player takes a direct drive to the basket (see Fig 4). Similar to Leg position, the only substantial differences in Movement variable relative frequencies are between the Youth competitions and the Senior competitions: there are more dribbles and/or cuts to the basket in Youth basketball. After adjustment, these differences decrease, but remain.

#### Shot success

**Fig 5:**
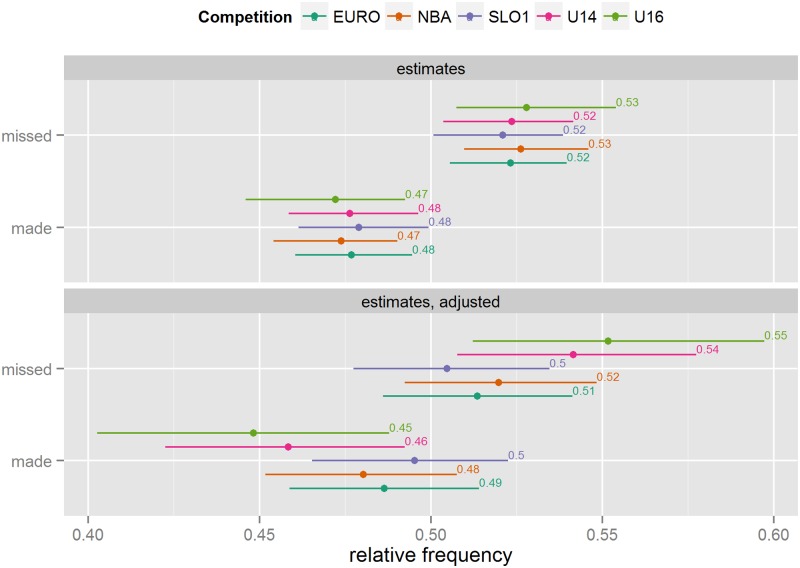
Shot success frequency estimates (unadjusted and adjusted for differences in situational variables).

There are no discernible differences between competitions in terms of overall shot success (see Fig 5). Adjusting the estimates results in a decrease in the estimated shot success for the Youth competitions, which is what we would expect, given that the average distance is much lower in Youth competitions. However, the overall variability of the success of a basketball shot (basically, a coin flip) prevents us from making a more accurate comparison across competitions.

### Fundamental differences between competitions

Table 2 compares the three models’ predictive accuracies. As we would expect, taking into account the situational variables substantially improves predictive accuracy. That is, Models 2 and 3 are substantially more accurate predictors that Model 1.

**Table 2:** Model evaluation and comparison.

	Model	elpd_WAIC_	Δ_Model3_	se(Δ)
Shot type	1	-5695.56	-1971.52	53.69
	2	-3883.55	-159.51	21.09
	3	-3724.04	-	-
Leg position	1	-2611.66	-1217.19	41.12
	2	-1752.21	-25.17	7.47
	3	-1727.04	-	-
Drive	1	-2792.23	-843.88	37.53
	2	-1972.84	-24.49	8.62
	3	-1948.35	0.00	0.00
Shot success	1	-3481.02	-182.39	19.27
	2	-3295.54	3.09	3.40
	3	-3298.63	-	-

The Δ_Model3_ column is the sum of the point-wise differences in performance between the model and Model 3 for the same target variable. The se(Δ) column is the standard error of this sum.

Model 2 assumes that situational variables have an effect on the target variables but that their effect (coefficients *β*) is the same across all competitions. Model 3 generalizes model two and allows competition-specific coefficients. However, the differences between using Model 3 and Model 2 in terms of predictive accuracy are not large. The only non-negligible difference is for Shot type, where most of the differences can be explained by the fact that Youth basketball players, especially U14, do not (can not) dunk or tip the ball in.

Our main objective was to estimate the (adjusted) relative frequencies, so we will not interpret individual coefficients. We only note that all estimated coefficients are in agreement with what we would expect from practical experience. Estimated coefficients with interpretations are provided for all Models 2 S2 Text. We opt not to report Model 3 coefficients, because these models are not substantially better than their corresponding Model 2. Furthermore, competition-specific coefficients are difficult to interpret.

## Discussion

As far as the investigated shot variables are concerned, all three Senior basketball competitions are surprisingly similar. NBA and Euroleague teams and players are superior to Slovenian Division 1 teams and players, so the lack of substantial statistical dissimilarities implies that players’ defensive and offensive abilities scale similarly if we move up or down in level of competition.

There are two discernible differences within the Senior competitions. First, in the NBA dunks are more frequent, and second, hook shots are less frequent compared to European basketball. Both can be, at least partially, attributed to better athleticism of NBA players, who are able to execute the more high-percentage dunk in more situations. However, the hook shot is a shot that is not only very difficult to block but also very difficult to alter (that is, it is also very difficult for the defender to interfere with this type of shot enough to cause the shooter to deviate from his typical execution of the shot and subsequently decreasing the likelihood of scoring) [21]. The hook shot has always played a role in basketball, especially for centres, so a lower relative frequency of this shot can also be partially explained by, at least in this respect, inferior technique of today’s NBA centres. Our results confirm the popular belief that the hook shot is disappearing from the NBA. Those that still utilize the shot in the NBA are typically European centres playing in the NBA (for example, Marc Gasol).

There are no discernible differences between U14 and U16, but U16 are in all observed variables more similar to the Senior competitions than U14, which is expected and can be attributed to their superior physical, tactical, and technical knowledge. The largest differences are between the Youth and the Senior competitions, but most of them can be explained with situational variables, in particular that the average Youth basketball shot is much closer to the basket. In the observed Youth games, more shots are executed jumping or standing on one leg (as opposed to two legs) and there is more dribbling or cutting to the basket. Shots near the basket are more successful. Allowing more such shots implies less effective 1-on-1 defending and closing down. While this might be effective in Youth basketball, it is not in Senior basketball, so during the transition from Youth to Senior level emphasis should be put on two-legged long-range above-head shots. Also, there are more uncategorised shot types (especially in U14), which can be attributed to the fact that these players are still developing proper technique and tactics.

We gain little in terms of predictive accuracy by allowing for competition-specific effects of situational variables. That is, while individual competitions may have different base preferences for shot types and technique, the effects of situational variables on shot selection appear to be consistent across all competitions. While this was not our main objective, the fitted statistical models also allowed us to estimate relative effectiveness of individual shot types and the effects of distance, transition, player type, etc… (see S2 Text for details). All results agree with expert knowledge.

We collected a substantial amount of data, however, they are still insufficient to illuminate more subtle differences between competitions, if they exist. More data would be needed for a more precise analysis, for shot success in particular. With the collected data, we could not analyse temporal aspects of the game. In particular, the connection between game pace (number of shots attempted in a unit time period, which also depends on how successful teams are at offensive rebounding), shots success, and shot selection. It is possible that differences in game pace or its in-game variability could explain some of the remaining differences in shot selection and shot success. However, we would require temporal data, including time left on the shot clock when the shot was attempted.

A little under 10% of all shots were left uncategorised (*other*). Some of these shots were unconventional shots made at the end of each quarter from a large distance as time was running out, however, a more detailed categorization of the remaining shots is necessary. Two important variables that have an effect on basketball shooting, but were not included in our study, are type of defence (man-to-man, zone) and the amount of pressure put on the shooter by the defence. We aim to address these issues as part of further work.

## Supporting Information

S1 Video A demonstration of an above head shot (two-legged). (MP4)

S2 Video A demonstration of a hook shot (two-legged). (MP4)

S3 Video A demonstration of a layup (one-legged). (MP4)

S4 Video A demonstration of a tip-in (two-legged). (MP4)

S5 Video A demonstration of a dunk (two-legged). (MP4)

S6 Video A demonstration of a layup (two-legged). (MP4)

S7 Video A demonstration of a hook shot (one-legged). (MP4)

S8 Video A demonstration of a layup, after running past (imaginary) defender. (MP4)

S9 Video A demonstration of an above head shot, after dribbling past (imaginary) defender. (MP4)

S1 Text List of games in the data set. Games are listed by competition with a brief explanation and date.
(PDF)

S2 Text Model coefficients. The estimated coefficients for Models 2 and 3 and every target variable. Brief interpretations are provided for Model 2 coefficients.
(PDF)

S1 Dataset The data we acquired and used in our analysis. The data are in csv format with headers.
(CSV)
