# Supplementary material for: Basketball Shot Types and Shot Success in Different Levels of Competitive Basketball
Source: PLoS One. 2015 Jun 3;10(6):e0128885. doi: 10.1371/journal.pone.0128885 (PMC4454648; doi:10.1371/journal.pone.0128885)
Supplement: S1 Text — Games are listed by competition with a brief explanation and date. (PDF) [file pone.0128885.s010.pdf]

NBA

All first games of the first round of the 2012/2013 NBA Playoffs:

Played on 20. 4. 2013:
Brooklyn Nets - Chicago Bulls
Denver Nuggets - Golden State Warriors
Los Angeles Clippers - Memphis Grizzlies
New York Knicks - Boston Celtics

Played on 21. 04. 2013:
Indiana Pacers - Atlanta Hawks
Miami Heat - Milwaukee Bucks
Oklahoma City Thunder - Houston Rockets
San Antonio Spurs - Los Angeles Lakers

EURO

All games of the first leg of the first round of the 2012/2013 Euroleague competition:

Played on 11. 10. 2012:
Mapooro Cantu - Union Olimpija Ljubljana
Fenerbahce Ulker - BC Khimki Moscow
Elan Chalon-Sur-Saone - Asseco Prokom
Unicaja Malaga - Maccabi Tel Aviv
Zalgiris Kaunas - Cedevita Zagreb
Olympiacos Piraeus - Laboral Kutxa Vitoria
CSKA Moscow - Lietuvos Rytas Vilnius

Played on 12. 10. 2012:
Real Madrid - Panathinaikos Athens
Montepaschi Siena - Alba Berlin
EA7 Emporio Armani Milan - Anadolu Efes
FC Barcelona Regal - Brose Baskets Bamberg
Besiktas Istanbul - Partizan MT:S Belgrade

SLO1

Ten regular season games from the 2012/2013 season of the top-tier Slovenian national competition:

Played on 24. 11. 2012:
Maribor Messer - Grosuplje
Rogaška Crystal - Elektra Šoštanj
Hopsi Polzela - Tajfun
Zlatorog Laško - LTH Castings Mercator

Played on 8. 12. 2012:
Zlatorog Laško - Grosuplje
Maribor Messer - Elektra Šoštanj
Rogaška Crystal - Slovan
Hopsi Polzela - Helios Domžale

Played on 16. 2. 2013:
Helios Domžale - Slovan

Played on 2. 3. 2013:
Tajfun - LTH Castings Mercator

U14

All games of the 2012 Slovenian U14 national competition Finals:

Played on 14. 12. 2012:
Krka - Slovan
Elektro Gorenjska prodaja - Dravograd

Played on 15. 12. 2012:
Krka - Elektro Gorenjska prodaja
Slovan - Dravograd

Played on 16. 12. 2012:
Dravograd - Krka
Elektro Gorenjska prodaja - Slovan

U16

All games of the 2012 Slovenian U16 national competition Finals:

Played on 6. 4. 2012:
Union Olimpija - Krka
Geoplin Slovan - Grosuplje

Played on 8. 4. 2012:
Union Olimpija - Geoplin Slovan
Krka - Grosuplje
