# Supplementary material for: Basketball Shot Types and Shot Success in Different Levels of Competitive Basketball
Source: PLoS One. 2015 Jun 3;10(6):e0128885. doi: 10.1371/journal.pone.0128885 (PMC4454648; doi:10.1371/journal.pone.0128885)
Supplement: S2 Text — The estimated coefficients for Models 2 and 3 and every target variable. Brief interpretations are provided for Model 2 coefficients. (PDF) [file pone.0128885.s011.pdf]

Model 2 estimated coefficients

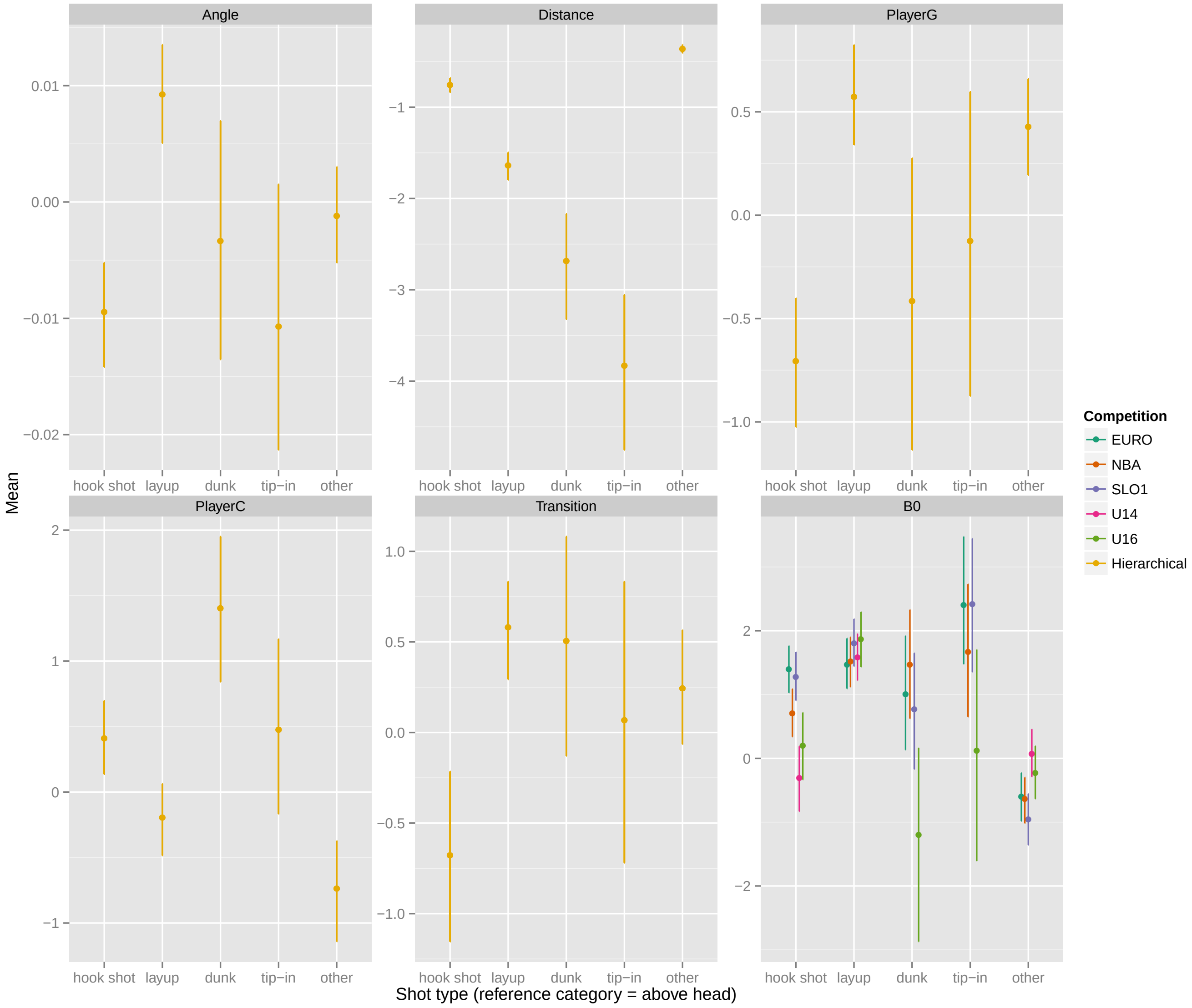

Figure 1: At higher angles, layups are more likely and hook shots less likely and angle little or no effect on the remaining shot types. Distance has a substantial effect on shot type - all shot types become less likely with distance (compared to the above head shot), which of course includes shot types that can only be attempted very close to the basket (dunk, tip-in). Compared to forwards, centres are more likely attempt hook shots, dunks and tip-ins, while less likely to attempt layups. Guards, on the other hand, are more likely to attempt layups and less likely to attempt the remaining three shot types. In transition, layups are more likely and hook shots are less likely than above the head shots. Note that U14 coefficients for dunk and tip-in were extremely low and were removed to facilitate visual comparison of other coefficients.

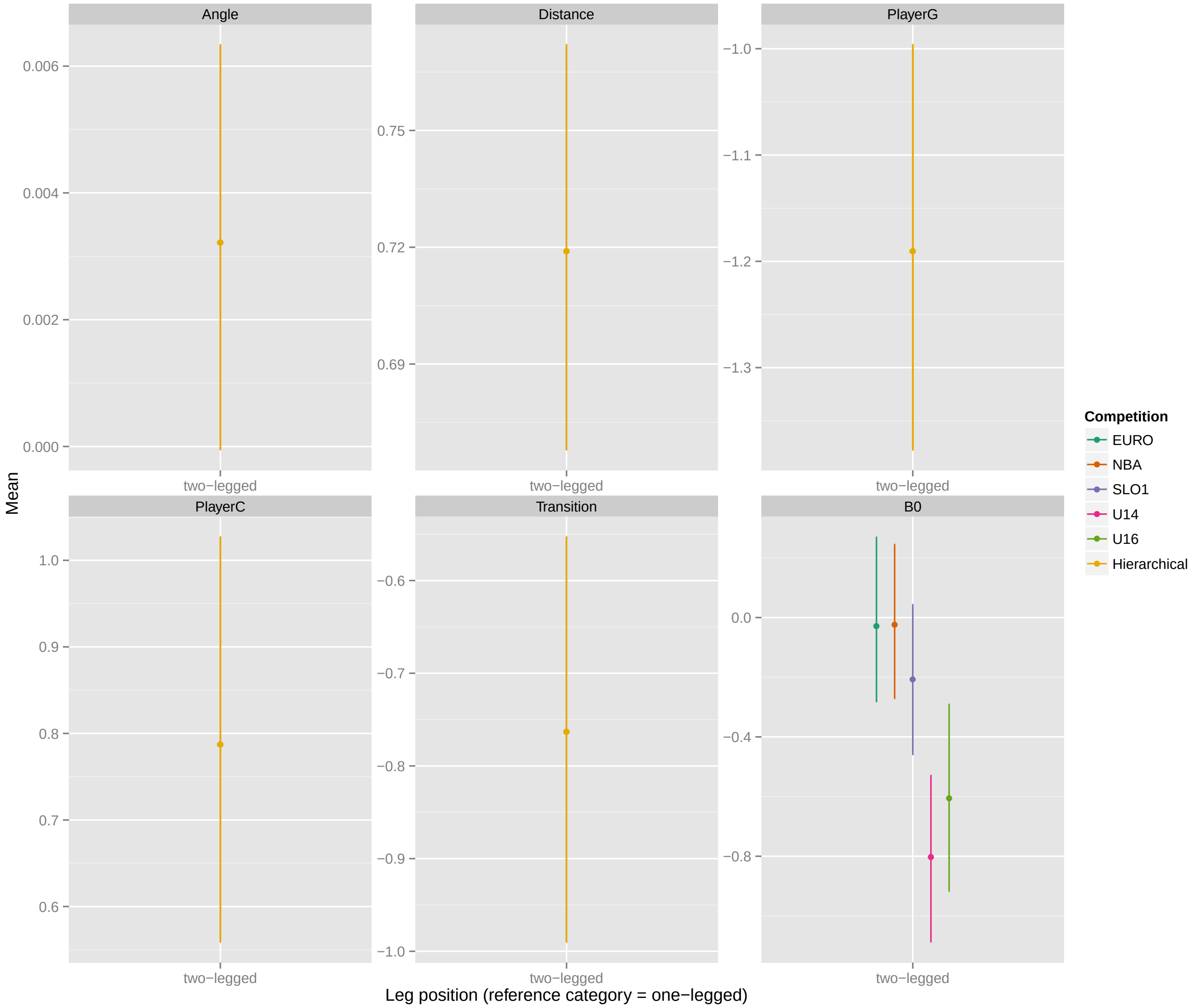

Figure 2: Angle has no substantial effect on leg position. With increasing distance, two-legged shots are more likely. In transition and with guards, one-legged shots become more likely, but less likely with centres.

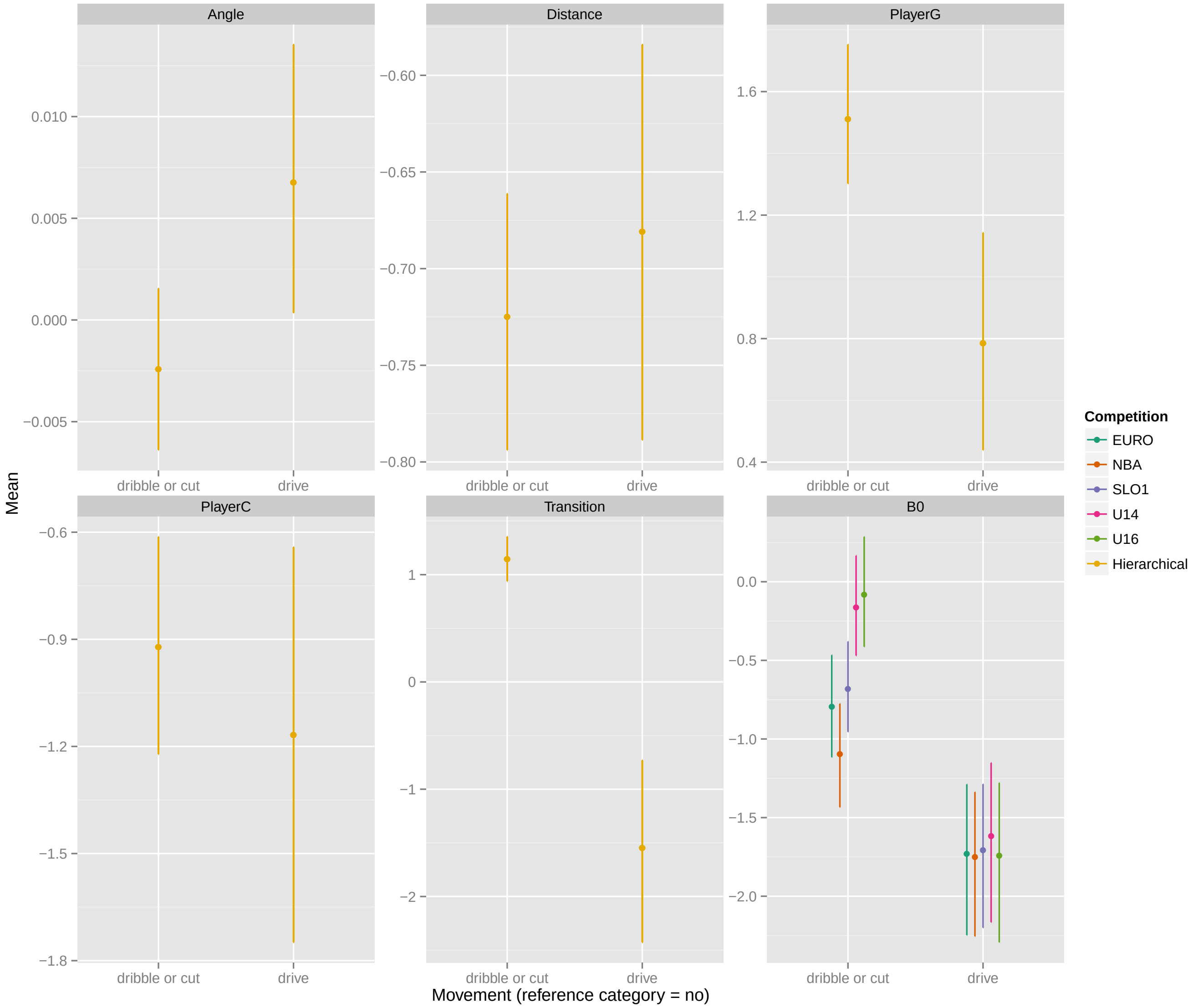

Figure 3: Angle again has little effect on movement before shot attempt while the likelihood of a dribble, cut or drive decreases with distance fro basket. Guards are more likely than forwards to attempt dribbles or cuts, while centres are less likely. Dribbles and cuts are more likely in transition than with a set defence.

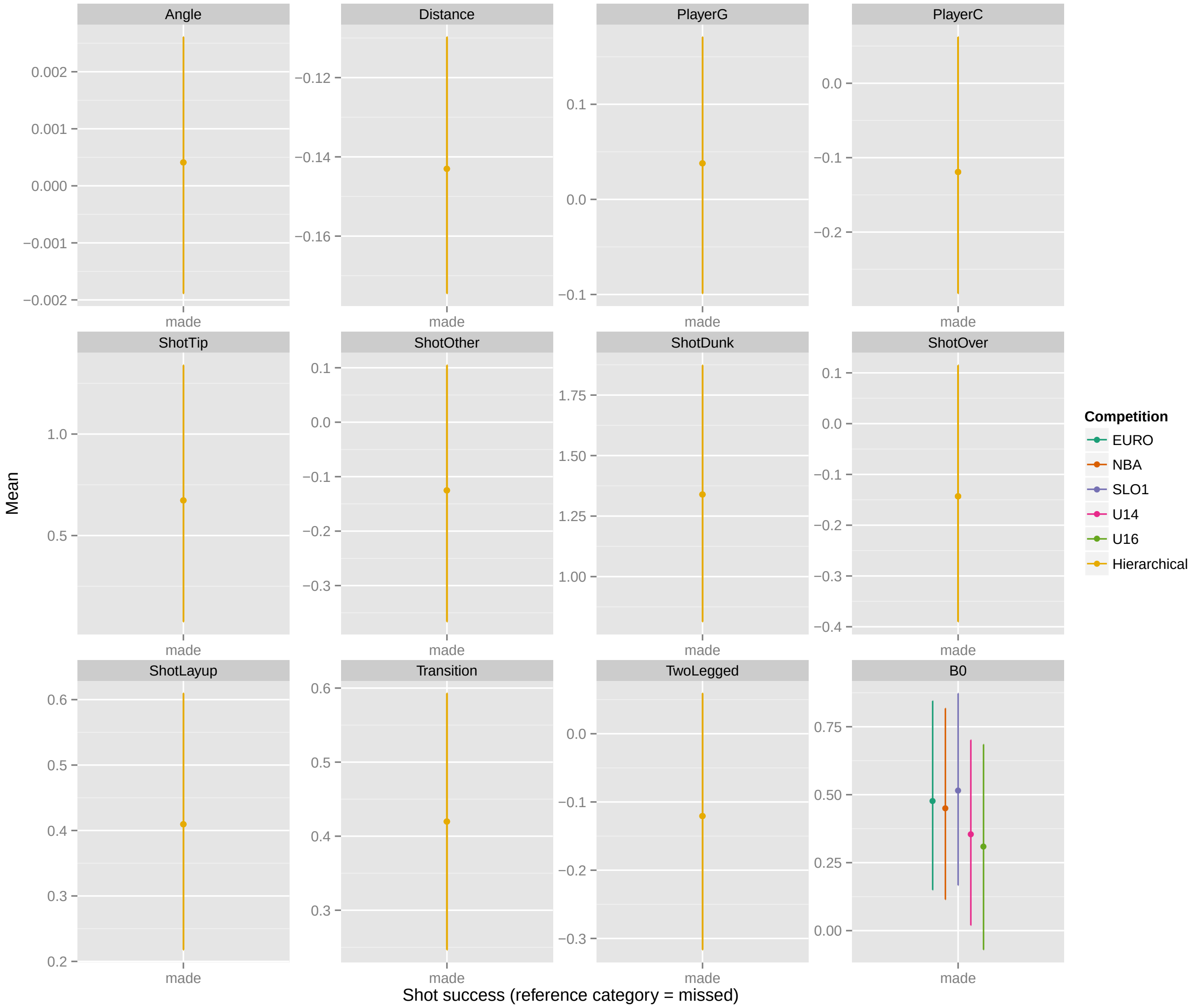

Figure 4: As we would expect, shot success decreases with distance but angle has no effect. Overall, guards are estimated to be more accurate than forwards and centres less accurate than forwards, but the differences are not discernible. Shots attempted in transition and layups are more successful, as are shots executed near the basket (tip-in, dunk).
